# Pre-Admission Statin Use and In-Hospital Severity of 2009 Pandemic Influenza A(H1N1) Disease

**DOI:** 10.1371/journal.pone.0018120

**Published:** 2011-04-25

**Authors:** Stephen J. Brett, Puja Myles, Wei Shen Lim, Joanne E. Enstone, Barbara Bannister, Malcolm G. Semple, Robert C. Read, Bruce L. Taylor, Jim McMenamin, Karl G. Nicholson, Jonathan S. Nguyen-Van-Tam, Peter J. M. Openshaw, the Influenza Clinical Information Network (FLU-CIN)

**Affiliations:** 1 Centre for Peri-operative Medicine and Critical Care Research, Imperial College Healthcare NHS Trust, London, United Kingdom; 2 Division of Epidemiology and Public Health, University of Nottingham, Nottingham, United Kingdom; 3 Department of Respiratory Medicine, Nottingham University Hospitals NHS Trust, Nottingham, United Kingdom; 4 Department of Health, Skipton House, London, United Kingdom; 5 Department of Women's and Children's Health, University of Liverpool, Liverpool, United Kingdom; 6 Department of Infection and Immunity, University of Sheffield, Royal Hallamshire Hospital, Sheffield, United Kingdom; 7 Department of Critical Care, Portsmouth Hospitals NHS Trust, Portsmouth, United Kingdom; 8 Health Protection Scotland, NHS National Services, Glasgow, United Kingdom; 9 Infectious Diseases Unit, University Hospitals of Leicester NHS Trust, Leicester Royal Infirmary, Leicester, United Kingdom; 10 Centre for Respiratory Infections, National Heart and Lung Institute, Imperial College, London, United Kingdom; University of Hong Kong, Hong Kong

## Abstract

**Background:**

Statins are drugs that are used to lower plasma cholesterol levels. Recently, contradictory claims have been made about possible additional effects of statins on progression of a variety of inflammatory disorders, including infections. We therefore examined the clinical course of patients admitted to hospital with 2009 pandemic influenza A(H1N1), who were or weren't taking statins at time of admission.

**Methods:**

A retrospective case-control study was performed using the United Kingdom Influenza Clinical Information Network (FLU-CIN) database, containing detailed information on 1,520 patients admitted to participating hospitals with confirmed 2009 pandemic influenza A(H1N1) infection between April 2009 and January 2010. We confined our analysis to those aged over 34 years. Univariate analysis was used to calculate unadjusted odds ratios (OR) and 95 percent confidence intervals (95%CI) for factors affecting progression to severe outcome (high dependency or intensive care unit level support) or death (cases); two multivariable logistic regression models were then established for age and sex, and for age, sex, obesity and “indication for statin” (e.g., heart disease or hypercholesterolaemia).

**Results:**

We found no statistically significant association between pre-admission statin use and severity of outcome after adjustment for age and sex [adjusted OR: 0.81 (95% CI: 0.46–1.38); n = 571]. After adjustment for age, sex, obesity and indication for statin, the association between pre-admission statin use and severe outcome was not statistically significant; point estimates are compatible with a small but clinically significant protective effect of statin use [adjusted OR: 0.72 (95% CI: 0.38–1.33)].

**Conclusions:**

In this group of patients hospitalized with pandemic influenza, a significant beneficial effect of pre-admission statin use on the in-hospital course of illness was not identified. Although the database from which these observations are derived represents the largest available suitable UK hospital cohort, a larger study would be needed to confirm whether there is any benefit in this setting.

## Introduction

In March 2009 the first human cases of a novel strain of pandemic influenza A(H1N1; hereafter pH1N1) virus of swine origin were reported from the United States; within three months global spread had occurred leading to the declaration of a pandemic by the World Health Organization. Whilst the majority of cases were mild or sub-clinical, about 1% of patients required admission to hospital [1], of whom about 15% required critical care support and an estimated 5–10% died [2]–[4]. Thus, even a relatively mild pandemic can have a considerable local and global impact, and inexpensive measures to reduce the effect of future pandemics remain an urgent priority.

Statins (three- hydroxy 3- methylgluteryl co enzyme A reductase inhibitors) are drugs indicated for control of plasma cholesterol. In addition, they have wide-ranging down-regulatory effects on inflammatory and immune mechanisms. Moreover, there is a body of circumstantial evidence that current treatment with statins may beneficially alter the natural history of infectious diseases, specifically reducing the likelihood of significant bacterial infection progressing to severe sepsis and septic shock [5]–[7]. It has been proposed that superadded bacterial infection was an important contributor to the substantial mortality associated with the 1918/19 pandemic [8]. In addition, a quarter of adults admitted to hospital and included in the Influenza Clinical Information Network database during the pH1N1 pandemic had clinically or radiographically apparent pneumonia [9], and there are reports that just under one third of such pneumonia cases had bacterial co-infection [10]–[12]. The anti-inflammatory effects of statins might have clinical benefits if severe disease is driven by over exuberant cytokine responses [13], as suggested in severe cases of both H5N1 and H1N1 influenza [14], [15]. It has thus been argued that during a period of high pandemic risk, the widespread administration of statins may be a clinically useful and cost effective public health measure [16], [17].

The Influenza Clinical Information Network (FLU-CIN) collected clinical, epidemiological and outcome data on patients admitted to participating UK hospitals with confirmed pH1N1 influenza infection. Data were also collected on medications taken by patients at the time of admission and on antibiotics, antivirals and steroids during hospital admission.

The aim of this retrospective database analysis was to establish whether there is evidence that patients who were taking statins at the time of hospital admission for pH1N1 influenza were less likely to die or require critical care support during their illness than patients not taking statins.

## Methods

Between April 2009 and January 2010, FLU-CIN collected clinical, epidemiological and outcome data on patients admitted to participating UK hospitals with confirmed pH1N1 influenza infection. Seventy five hospitals in 31 cities or towns provided data. The details of data collection and the overall findings from the first wave of the 2009 pandemic have been described elsewhere [2].

Briefly, pH1N1 influenza infection was diagnosed by a positive reverse transcriptase polymerase chain reaction (PCR) result from respiratory samples obtained during the admission episode. A dataset was collected by specifically trained data collectors which included demography, acute and long-term medications, clinical observations, clinical course and outcome. FLU-CIN was a public health surveillance project for which the Ethics and Confidentiality Committee of the National Information Governance Board for Health and Social Care in England approved the collection, storage and use of personal data without the need for individual participant consent. This report represents a secondary analysis of the entire FLU-CIN database which includes data on patients from both waves of the UK pH1N1 experience.

### Patients

The entire FLU-CIN database included 1,520 patients, of which 601 were from the first wave (25^th^ April to 31^st^ August 2009), and 919 patients from the second (1^st^ September–27^th^ January 2010). Patients aged less than 35 years were excluded from this analysis, since few young people use statins, all others were included (n = 571). Cases were defined as patients who suffered a severe outcome (critical illness or death) whilst controls were those who did not.

### Analysis and statistical methodology

We used a case-control analysis to investigate the association between pre-admission statin use and severe outcome from influenza infection. Our exposure variable was ‘pre-admission statin use (yes/no)’ as recorded in the case note current drug history. For the purpose of the analysis we did not categorise statin use by type, duration or dosage. We defined ‘severe outcome’ (cases) as those patients requiring admission to level 2 (high dependency unit) or level 3 (intensive care unit) or who died (Data S1).

We used an *a priori* conceptual framework to determine potential confounding variables in the multivariate logistic regression rather than a statistical approach. We adjusted for age, sex, physician-defined obesity and ‘indication for statin’ (this binary variable was coded as ‘yes’ if the subject had any one of the following recorded in their case notes: heart disease- predominantly ischaemic heart disease and cardiac failure, cerebrovascular disease, hypercholesterolemia). This latter variable was developed to ameliorate the potential for “confounding by indication”, as statin use is associated with co-morbid states which contribute to additional individual clinical risk for worse outcomes in many clinical situations.

We used univariate analysis to calculate unadjusted odds ratios (OR) and 95 percent confidence intervals (95% CI), then two multivariable logistic regression models to calculate adjusted ORs and 95%CIs. Model 1 adjusted for age and sex, and model 2 for obesity and ‘indication for statin’ in addition to age and sex. We also carried out a number of sensitivity analyses: to explore level 2/3 admission and death separately as outcome measures, to look for a healthy user effect, and to determine impact of other co-morbidities not associated with statin use. We used Stata (version 11) for all statistical analyses

## Results

Our study sample comprised 571 patients aged 35 years and above (44% male, mean age: 52 years ±11.8) of whom 94/571 (16.5%; Table 1) had been taking statins at the time of hospital admission and 121/571 (21.2%) progressed to severe outcome; the profile of statins recorded was simvastatin 62%, atorvastatin 30%, rosuvastatin 5%, pravastatin 2%, unspecified 1%. Table 1 summarizes patient characteristics associated with severe outcomes in influenza patients. Women appeared to be at a lower risk of severe outcome than men [crude OR: 0.45 (95% CI: 0.30–0.68)]. As reported previously [14], C-reactive protein levels higher than 30 mg/l were associated with severe outcomes [crude OR: 3.28 (95% CI: 1.27–8.25)] and the likelihood of severe outcome increased further with levels greater than 100 mg/l [crude OR: 15.27 (95% CI: 6.06–38.43)]. We failed to find a statistically significant association between pre-admission statin use and severe outcome [crude OR: 0.93 (95% CI: 0.46–1.44)].

**Table 1:** Univariate analysis of factors influencing severe outcomes in pH1N1 patients, ages 35 years and above (n = 571).

Patient characteristic	Category	Cases (severe outcomes) (n = 121) (%)	Controls (n = 450) (%)	Unadjusted OR (95% CI)	P value
**Statins**	No	102 (84.3)	375 (83.3)	1.00	0.800
	Yes	19 (15.7)	75 (16.7)	0.93 (0.54–1.61)	
**Age group**	35–44	41 (33.9)	173 (38.4)	1.00	
	44–54	33 (27.3)	134 (29.8)	1.04 (0.63–1.73)	
	55–64	30 (24.8)	85 (18.9)	1.49 (0.87–2.55)	
	65–74	14 (11.6)	41 (9.1)	1.44 (0.72–2.89)	
	≥75	3 (2.5)	17 (3.8)	0.74 (0.21–2.66)	*P trend = 0.318*
**Sex**	Male	72 (59.5)	180 (40.0)	1.00	
	Female	49 (40.5)	270 (60.0)	**0.45 (0.30–0.68)**	**<0.001**
**Ethnicity**	White	57 (47.1)	196 (43.6)	1.00	
	Other	26 (21.5)	136 (30.2)	0.66 (0.39–1.10)	0.109
	Missing	38 (31.4)	118 (26.2)	1.11 (0.69–1.77)	0.671
**Statin indication**	No	103 (85.1)	393 (87.6)	1.00	
	Yes	18 (14.9)	57 (12.6)	1.21 (0.68–2.14)	0.518
**C-reactive protein (mg/l)**	<30	6 (5.0)	105 (23.3)	1.00	
	31–99	21 (17.4)	112 (24.9)	**3.28 (1.27–8.45)**	**0.014**
	>100	41 (33.9)	47 (10.4)	**15.27 (6.06–38.43)**	**<0.001**
	Missing	53 (43.8)	53 (43.8)	-	
**Obese**	No	111 (91.7)	423 (94.0)	1.00	
	Yes	10 (8.3)	27 (6.0)	1.41 (0.66–3.00)	0.371
**Smoking**	No	54 (44.6)	227 (50.4)	1.00	
	Yes	36 (29.8)	104 (23.1)	1.46 (0.90–2.35)	0.127
	Missing	31 (25.6)	119 (26.4)		
**Cardiovascular disease**	No	83 (68.6)	339 (75.3)	1.00	
	Yes	38 (31.4)	111 (24.7)	1.40 (0.90–2.17)	0.135
**Cerebrovascular disease**	No	121 (100.0)	446 (98.9)	1.00	
	Yes	0 (0.0)	5 (1.1)	-*	
**Diabetes**	No	102 (84.3)	395 (87.8)	1.00	
	Yes	19 (15.7)	55 (12.2)	1.34 (0.76–2.35)	0.313
**Immunocompromised status**	No	115 (95.1)	433 (96.2)	1.00	
	Yes	6 (4.9)	17 (3.8)	1.33 (0.51–3.45)	0.559
**Charlson comorbidity index score**	0	47 (38.8)	164 (36.4)	1.00	
	1–2	62 (51.2)	225 (50.0)	0.96 (0.63–1.48)	0.858
	3–5	12 (9.9)	57 (12.7)	0.74 (0.36–1.48)	0.389
	>5	0 (0.0)	4 (0.9)	-	
**Length of hospital stay**	<2 days	2 (1.7)	60 (13.3)	1.00	
	≥2 days	66 (54.6)	348 (77.3)	**5.69 (1.36–23.85)**	**0.017**
	Missing	53 (43.8)	42 (9.3)	-	

Note: Statistically significant results in bold.

(*could not be calculated because of insufficient data).

Table 2 presents the results of the multivariable logistic regression. We found no statistically significant association between pre-admission statin use and severe outcome after adjustment for age and sex [adjusted OR: 0.81 (95% CI: 0.46–1.38)]. Even after adjustment for age, sex, obesity and indication for statin, the association between pre-admission statin use and severe outcome failed to reach statistical significance although the point estimates are compatible with a clinically significant protective effect [adjusted OR: 0.72 (95% CI: 0.38–1.33)].

**Table 2:** Multivariable analysis: association between pre-admission statin use and severe outcomes in pH1N1 influenza.

Exposure		Unadjusted odds ratio (95% CI)	Model 1: Adjusted odds ratio (95% CI)	Model 2: Adjusted odds ratio (95% CI)
**Pre-admission statin use**	No	1.00	1.00	1.00
	Yes	0.93 (0.46–1.44)	0.81 (0.46–1.38)	0.72 (0.38–1.33)
**Age-group (years)**	35–44	1.00	1.00	1.00
	45–54	1.04 (0.63–1.73)	1.05 (0.62–1.76)	1.06 (0.63–1.79)
	55–64	1.49 (0.87–2.55)	1.48 (0.85–2.57)	1.50 (0.86–2.62)
	65–74	1.44 (0.72–2.89)	1.44 (0.70–2.99)	1.48 (0.71–3.09)
	≥75	0.74 (0.21–2.66)	0.64 (0.18–2.32)	0.64 (0.17–2.33)
**Sex**	Male	1.00	1.00	1.00
	Female	**0.45 (0.30–0.68)**	**0.45 (0.30–0.68)**	**0.45 (0.29–0.67)**
**Statin indication**	No	1.00		1.00
	Yes	1.21 (0.68–2.14)	-NA-	1.23 (0.65–2.33)
**Obese**	No	1.00		1.00
	Yes	1.41 (0.66–3.00)	-NA-	1.66 (0.75–3.67)

Model 1: adjusted for a priori confounders age and sex.

Model 2: adjusted for age, sex, indication for statin and obesity.

A number of sensitivity analyses were performed. First, level 2/3 admissions and death were considered as separate outcomes. After adjusting for age, sex, obesity and indication for statins, pre-admission statin use was associated with a decreased risk of death [adjusted OR: 0.64 (95% CI: 0.25–1.65)] and level 2/3 admission [adjusted OR: 0.66 (95% CI: 0.33–1.29)] but again, these results did not reach statistical significance. A second analysis was conducted to explore whether statin use was simply a marker for the ‘healthy user effect’ (implying that statin users were healthier than non-users at baseline thereby explaining the observed decrease in severe outcomes). However, conditions that are common ‘indications for statin’ (hypercholesterolemia, cerebrovascular disease, cardiovascular disease) are serious comorbidities that can generally affect health in an unfavorable way. In our cohort, we found that statin users were more likely to have a condition that would qualify as a ‘statin indication’ (unadjusted OR 45.32, 95% CI: 25.76–79.73, p<0.001). Finally, we added a further balancing term to Model 2 which was the presence or absence of a co-morbidity not normally *directly* associated with statin use (hypertension; asthma; chronic lung, liver or renal disease; diabetes or other metabolic disease; chronic neurological disease). This did not affect the outcome (adjusted OR 0.71 (95% CI: 0.38–1.33).

## Discussion

In this retrospective secondary analysis, we were unable to demonstrate a statistically significant impact of statins on the progression of influenza to a severe outcome in patients hospitalized during the UK 2009 pandemic. Whilst not achieving significance, our effect point estimates are consistent with previously published positive reports [5]–[7], [18], [19]. We are therefore unable to confidently exclude a beneficial effect of statin use in patients with severe influenza.

There is evidence from sepsis research that statins ameliorate inflammatory and immunological function by so-called pleiotropic effects on leukocyte-endothelial interaction, intra- and inter-cellular signaling, inflammatory gene transcription, haem-oxygenase expression and expression of MHC class II antigens [13]. The net effect is thought to be a general “damping-down” of inflammatory state. There is some modest supporting evidence from a trial of simvastatin in suspected or proven bacterial infection; reductions in tumor necrosis factor-α and interleukin-6 were demonstrated in the statin treated arm [20]. A variety of epidemiological studies have generally been further supportive of this hypothesis, suggesting that statins may have a beneficial effect in modifying severe responses to infectious insults in general populations [5]–[7], and chronic renal failure patients [21].

A detailed review of these and other individual studies lies outside the scope of this paper but two thorough meta-analyses have been published recently [22], [23]. Search strategies differed somewhat and spanned different epochs, but the conclusions were broadly similar. Tleyjeh and colleagues concluded that there was evidence that statins appeared overall beneficial in the prevention and treatment of infections [22], although the authors identified weak effects in some studies and some evidence of publication bias. Janda and colleagues more recent synthesis concluded that there was a protective effect of statins in patients with sepsis and/or other severe infections [23]; the authors felt their results were limited by the cohort-nature of some included studies and trial outcome heterogeneity.

Several important studies relevant to the current report deserve brief discussion [18]–[19], [24]–[26]; a number of these were community-focused and thus not included in the meta-analyses. As respiratory impairment is a key factor in progression to severe outcome, the effect on clinically important pneumonia, which may of course be bacterial or viral is a key question. In a community cohort study of 3,681 patients with pneumonia [18], Myles and colleagues observed that current statin use was associated with a 67% reduction in 30 day mortality (adjusted hazard ratio 0.33, 95%CI 0.19–0.58), and a 55% reduction in long term mortality (adjusted hazard ratio 0.45, 95%CI 0.32–0.62). In a related report from the same group [19], current statin use was associated with a reduced risk of developing pneumonia (odds ratio 0.78, 95%CI 0.65–0.94).

By contrast, a matched case control study of people aged over 65 years [24], did not demonstrate a clear positive benefit. The investigators identified 1,125 cases of pneumonia and 2,235 matched controls; current statin users represented 16.1% of cases and 14.6% of controls (adjusted odds ratio 1.26, 95%CI 1.01–1.56). Similarly, statin use was present in 17.2% of cases admitted to hospital and 14.2% of matched controls (adjusted odds ratio 1.61, 95%CI 1.08–2.39).

A study of 3,415 hospitalized patients with pneumonia appeared to show a reduction in critical care admission and mortality [25] (included in both meta-analyses) in patients previously treated with statins. However, this beneficial effect disappeared after adjustment for confounders and statin use may even have been associated with potential harm. A long term community study over several winters [26], including data on nearly a third of a million patient years, failed to demonstrate an impact of statin treatment on the incidence of respiratory infection, although there was a significant reduction in urinary tract infections.

What these epidemiological studies lack are robust microbiological data differentiating bacterial and viral causes of pneumonia. A report of the Australasian critical care pandemic influenza pH1N1 influenza cohort (n = 689) reported a viral pneumonitis rate of 48.8%, and a secondary bacterial pneumonia rate of 20.3% [11]. A recent Canadian study of critically ill pandemic influenza patients reported a secondary bacterial pneumonia rate of 24.4% [12]. Acute respiratory failure in patients without a clear diagnosis of viral pneumonitis or bacterial pneumonia may be due to acute lung injury associated with multiple organ failure, exacerbation of underlying respiratory or cardiac disease, fluid overload or other causes. The proportion of patients whose disease progression is due to bacterial superinfection may be a critical variable in understanding the effect of statins, since uncomplicated viral lung disease may be less affected by statin use.

There are a number of possible explanations for our findings. First, the benefits of statins may be too modest for our study to detect. (type 2 error). The event rate (progression to severe outcome) for the statin naïve group (n = 477) was 21.4%, and 20.2% for prior statin treated patients (n = 94; p = 0.8).

This study was an opportunistic study arising from access to the pandemic influenza surveillance database and we did not perform prior sample size calculations. We decided to carry out a post-hoc power calculation based on estimates of statin effect from previous reports [18], [19]. Assuming a probability of statin use of 0.17 and a 30 percent decrease in severe outcomes for influenza with statins, we estimated that a future case control study would require 637 cases and 2,548 controls to meet the standard criteria of 80 percent power. This suggests that our present study may have been underpowered, which could explain why we did not observe a statistically significant effect. Interestingly, our point estimate (0.72) was very close to previous study estimates of the protective effect of statins on pneumonia-related mortality (although clearly this could have been a chance finding). A second limitation is that our analysis is confined to hospitalized cases. If statins reduce the severity of influenza in non-hospitalized patients, cases reaching the threshold for hospitalization despite statin use may have an in-hospital course similar to that seen in non-users of statins. Third, we did not record whether statin therapy continued after admission to hospital. We cannot therefore determine whether stopping or continuing statin therapy affected outcome. Last, there may be no or little class effect of statins on disease severity during influenza infection; the majority of patients were taking simvastatin, the dataset was not large enough to explore for effects of individual statin drugs. We do not know what proportion of patients progressed to severe outcome because of pathological processes affected by statins and how many patients progressed for other reasons. Our cohort was not large enough to justify subgroup analyses, and we are therefore unable to exclude beneficial effects in certain patient groups.

In conclusion, we were unable to show a beneficial effect of prior statin use on the in-hospital course of illness in patients admitted with pH1N1 influenza. Although the database from which these observations are derived represents the largest UK hospital cohort with detailed pre-admission data, it is possible that a significant beneficial effect has been missed and that a larger study could reveal a clinically significant benefit from statin administration.

## Supporting Information

Data S1 **Levels of care.**
(DOCX)
