# Supplementary material for: Pre-Admission Statin Use and In-Hospital Severity of 2009 Pandemic Influenza A(H1N1) Disease
Source: PLoS One. 2011 Apr 25;6(4):e18120. doi: 10.1371/journal.pone.0018120 (PMC3081811; doi:10.1371/journal.pone.0018120)
Supplement: Data S1 — Levels of care. (DOCX) [file pone.0018120.s001.docx]

**Supporting Information: Data S1**

**Levels of Care**

**Level 0:** Patients whose care needs can be met through normal ward care; **Level 1:** Patients at risk of deteriorating or recently relocated from higher levels of care whose needs can be met on an acute ward with additional advice and support from the critical care team; **Level 2:** Patients requiring more detailed observation or intervention including support for a single failing organ system and those ‘stepping down’ from higher levels of care – High Dependency Unit; **Level 3:** Patients requiring advanced respiratory support alone or basic respiratory support together with support of at least two organ systems. This includes all complex patients requiring support for multi-organ failure – Intensive Care Unit.
